# Association of an Accelerated High-Sensitivity Cardiac Troponin-Anchored Care Pathway With 30-Day Mortality and Intensive Care Outcomes in Acute Coronary Syndrome: A Retrospective Cohort Study

**DOI:** 10.7759/cureus.112645

**Published:** 2026-07-14

**Authors:** Yousaf Saeed, Muhammad Athif Akram, Muhammad Nauman Shahid

**Affiliations:** 1 Department of Internal Medicine, University at Buffalo, State University of New York, Buffalo, USA; 2 Department of Anesthesiology and Intensive Care, Abu Umara Medical and Dental College/Ali Fatima Teaching Hospital, Lahore, PAK; 3 Department of Biological Sciences, Ghurki Trust Teaching Hospital, Lahore, PAK

**Keywords:** acute coronary syndrome, cardiogenic shock, early management, high-sensitivity cardiac troponin, intensive care unit, mortality, myocardial infarction

## Abstract

Background: Acute coronary syndrome causes substantial short-term mortality, cardiovascular complications, and intensive care use. High-sensitivity cardiac troponin within an accelerated care pathway may support earlier recognition and management.

Objective: To assess the association between an accelerated high-sensitivity cardiac troponin-anchored care pathway and 30-day mortality and intensive care outcomes in acute coronary syndrome.

Methods: This retrospective cohort study was conducted at the Department of Internal Medicine, University at Buffalo, New York, from April 2024 to April 2025. Records of 200 patients were reviewed: 100 managed through the accelerated pathway and 100 receiving standard care. The pathway was defined as definitive clinical action within three hours based on troponin findings, clinical assessment, and electrocardiography. The primary outcome was 30-day all-cause mortality; secondary outcomes were exploratory.

Results: Measured baseline characteristics did not differ significantly. Time to definitive decision was shorter with the accelerated pathway (2.1 ± 0.6 vs. 5.7 ± 1.8 hours; p<0.001). Thirty-day mortality was 7.0% vs. 16.0%, with an absolute risk difference of −9.0 percentage points (95% confidence interval: −17.8 to −0.2; p=0.046). Acute heart failure, malignant arrhythmias, cardiogenic shock, intensive care unit admission, and hospital stay were lower in exploratory unadjusted analyses. Exploratory multivariable analysis showed an association with lower 30-day mortality (adjusted odds ratio: 0.40; 95% confidence interval: 0.16-0.98; p=0.045).

Conclusion: The accelerated pathway was associated with lower observed mortality and fewer short-term complications. Causal conclusions cannot be drawn because of the retrospective, non-randomized design and potential residual confounding.

## Introduction

Acute coronary syndrome is a major cardiovascular emergency that includes ST-segment elevation myocardial infarction, non-ST-segment elevation myocardial infarction, and unstable angina [1]. It remains an important cause of emergency department visits, coronary care admissions, intensive care utilization, prolonged hospitalization, and short-term mortality. Early recognition and timely treatment are essential because persistent myocardial ischemia may progress to infarct extension, acute left ventricular dysfunction, malignant arrhythmias, cardiogenic shock, cardiac arrest, and death [2].

Diagnosing acute coronary syndrome may be challenging, particularly in patients with non-ST-segment elevation myocardial infarction or unstable angina [3]. Although chest pain is the predominant presentation, older adults, women, patients with diabetes mellitus, and those with chronic kidney disease may present with dyspnea, fatigue, nausea, epigastric discomfort, syncope, diaphoresis, or generalized weakness. Electrocardiographic changes may also be transient, non-specific, or absent in non-ST-segment elevation acute coronary syndrome. These factors may delay risk stratification and definitive management in patients at risk of adverse outcomes [4].

Cardiac troponin is the principal biomarker used to detect myocardial injury in patients with suspected acute coronary syndrome [5]. High-sensitivity cardiac troponin assays detect lower circulating concentrations and permit earlier assessment than conventional assays. Serial testing allows evaluation of baseline concentrations and dynamic changes over time, supporting differentiation between acute myocardial injury and chronic troponin elevation [6].

However, an elevated high-sensitivity troponin concentration alone does not establish acute myocardial infarction [7]. Troponin elevation may also occur in acute heart failure, severe hypertension, tachyarrhythmia, pulmonary embolism, sepsis, renal impairment, myocarditis, and other critical illnesses. High-sensitivity troponin results should therefore be interpreted alongside ischemic symptoms, electrocardiographic findings, serial biomarker changes, hemodynamic status, renal function, echocardiographic findings where required, and the overall clinical risk profile [8,9].

The clinical value of high-sensitivity troponin depends not only on earlier detection of myocardial injury but also on its timely integration into clinical decision-making [10]. An accelerated high-sensitivity troponin-anchored care pathway may facilitate early cardiology assessment, evidence-based antithrombotic and lipid-lowering therapy, continuous cardiac monitoring, admission to an appropriate monitored setting, echocardiography, invasive coronary evaluation, and revascularization where indicated. These components constitute a coordinated acute coronary syndrome care pathway rather than an isolated troponin intervention [11].

Delays may occur during hospital presentation, electrocardiography, troponin sampling and reporting, interpretation of serial results, specialist assessment, monitored-unit transfer, and access to coronary angiography or revascularization [12]. These delays may be particularly relevant in non-ST-segment elevation myocardial infarction, where management frequently depends on serial biomarkers and overall risk assessment rather than persistent ST-segment elevation [13]. Social determinants, including socioeconomic circumstances, insurance status, transportation, health literacy, and access to emergency and specialist services, may also influence presentation and treatment timing and contribute to cardiovascular disparities [14].

Although high-sensitivity troponin assays have improved early assessment, biomarker detection alone may not influence outcomes unless abnormal or changing results lead to an organized management response. Most available studies have focused on diagnostic performance or myocardial infarction exclusion, whereas evidence evaluating mortality and intensive care-related outcomes within an accelerated troponin-anchored care pathway remains limited in routine clinical practice [15,16]. The present study therefore contributes by examining 30-day mortality together with acute heart failure, malignant arrhythmias, cardiogenic shock, intensive care unit admission, and hospital stay within a single coordinated pathway [17].

Therefore, this retrospective observational cohort study aimed to assess the association between an accelerated high-sensitivity cardiac troponin-anchored care pathway and 30-day all-cause mortality in patients with acute coronary syndrome. The secondary objective was to compare in-hospital mortality, recurrent myocardial infarction, acute heart failure, malignant arrhythmias, cardiogenic shock, intensive care unit admission, and hospital stay between patients managed through the accelerated pathway and those receiving standard care. We hypothesized that accelerated-pathway management would be associated with lower 30-day mortality and fewer short-term cardiovascular and intensive care complications [18,19].

## Materials and methods

Study design and setting

This retrospective observational cohort study was conducted at the Department of Internal Medicine, University at Buffalo, Buffalo, New York, from April 2024 to April 2025. The University at Buffalo served as the primary clinical research center. Ghurki Trust Teaching Hospital, Lahore, Pakistan, affiliated with Lahore University of Biological and Applied Sciences (UBAS), a project of Lahore Medical and Dental College (LMDC), provided academic collaboration. The study evaluated the association between an accelerated high-sensitivity cardiac troponin-anchored care pathway and 30-day mortality and intensive care outcomes in patients with acute coronary syndrome.

Study population and grouping

Medical records of 200 consecutive adults with suspected or confirmed acute coronary syndrome were reviewed. Diagnosis was based on clinical presentation, electrocardiographic findings, serial high-sensitivity cardiac troponin measurements, and the treating physician’s final assessment. Patients with ST-segment elevation myocardial infarction, non-ST-segment elevation myocardial infarction, or unstable angina were included. In patients with ST-segment elevation myocardial infarction, reperfusion decisions were primarily electrocardiography-driven and were not delayed while awaiting troponin results.

Patients were categorized according to the timing of documented definitive management. The accelerated-pathway group included 100 patients who received definitive clinical action within three hours of hospital presentation based on high-sensitivity troponin findings, clinical assessment, and electrocardiography. The standard-care group included 100 patients managed after three hours or through routine clinical assessment. Group allocation reflected routine clinical practice and was not randomized.

Because this was a retrospective record-based study, no prospective sample size calculation was performed. All consecutive eligible records with complete in-hospital and 30-day outcome data during the study period were considered, yielding a final analytic sample of 200 patients, with 100 patients in each group.

Inclusion and exclusion criteria

Patients aged 18 years or older with symptoms suggestive of acute coronary syndrome, including chest discomfort, dyspnea, diaphoresis, syncope, palpitations, nausea, or unexplained fatigue, were eligible. Inclusion required high-sensitivity cardiac troponin testing at presentation and complete in-hospital and 30-day outcome data.

Patients with myocardial injury attributable to myocarditis, pulmonary embolism, sepsis, severe trauma, recent major surgery, severe anemia, uncontrolled tachyarrhythmia without acute coronary syndrome, advanced malignancy, or dialysis-dependent end-stage renal disease were excluded. Records with missing essential clinical variables, uncertain acute coronary syndrome diagnosis, duplication, or incomplete 30-day follow-up were also excluded. A complete-case approach was used, and no statistical imputation of missing data was performed.

Accelerated high-sensitivity troponin-anchored care pathway

High-sensitivity cardiac troponin was measured at hospital presentation and repeated approximately three hours after the initial sample or earlier when clinically indicated. Additional measurements were performed when symptoms persisted, clinical status changed, or diagnostic uncertainty remained. Results were interpreted using the assay-specific 99th-percentile upper reference limit and the presence of a clinically meaningful serial rise or fall according to the institutional laboratory protocol. Troponin findings were interpreted alongside ischemic symptoms, electrocardiographic changes, renal function, hemodynamic status, and overall clinical risk.

Accelerated-pathway management was defined as documented definitive clinical action within three hours of hospital arrival. Management could include early internal medicine or cardiology assessment, antiplatelet therapy, anticoagulation where indicated, high-intensity statin therapy, continuous cardiac monitoring, monitored-unit admission, echocardiography, coronary angiography, reperfusion therapy, percutaneous coronary intervention, or escalation of care according to clinical need. These measures represented an accelerated acute coronary syndrome care bundle rather than the isolated effect of troponin testing.

Data collection and outcome assessment

Data were extracted from medical records using a standardized clinical proforma. Recorded variables included demographic characteristics, cardiovascular risk factors, comorbidities, presenting features, time from symptom onset to presentation, vital signs, Killip class, electrocardiographic findings, acute coronary syndrome subtype, serial troponin measurements, renal function, hemoglobin, blood glucose, echocardiographic findings, treatment received, time to cardiology review, time to definitive decision, coronary angiography, revascularization, intensive care unit admission, and in-hospital complications.

Acute coronary syndrome subtype and clinical outcomes were determined from the treating physician’s final diagnosis, electrocardiographic and laboratory findings, imaging reports, discharge summaries, and follow-up documentation. Records with uncertain diagnoses or inadequately documented outcomes were excluded from the analysis.

Left ventricular ejection fraction was recorded from available echocardiographic reports. Because complete and comparable measurements were unavailable for all patients in both groups, left ventricular ejection fraction was not included in the principal comparative analysis.

The primary outcome was 30-day all-cause mortality. Secondary outcomes included in-hospital mortality, recurrent myocardial infarction, acute heart failure, malignant arrhythmias, cardiogenic shock, cardiac arrest, intensive care unit admission, vasopressor or inotropic use, ventilatory support, intensive care unit stay, and total hospital stay. Secondary outcomes were considered exploratory.

Follow-up and statistical analysis

Thirty-day outcomes were obtained from hospital records, discharge summaries, outpatient documentation, and telephone follow-up where required. Mortality and major clinical outcomes were documented for all included patients.

Data were analyzed using SPSS version 27.0 (IBM Corp., Armonk, NY). Continuous variables were presented as mean ± standard deviation and compared using the independent-samples t-test. Categorical variables were presented as frequencies and percentages and compared using the chi-square test or Fisher’s exact test. Crude effect estimates with 95% confidence intervals were reported where applicable.

Multivariable logistic regression was performed as an exploratory analysis of factors associated with 30-day mortality. The model included accelerated-pathway management, age above 65 years, diabetes mellitus, hypertension, ST-segment elevation myocardial infarction presentation, Killip class III-IV, delayed presentation beyond six hours, and cardiogenic shock. Because only 23 deaths occurred, adjusted estimates were interpreted cautiously because of the potential for model overfitting. Chronic kidney disease was assessed descriptively but was not included in the final model because of the limited event count.

No propensity-score, penalized-regression, landmark, or acute coronary syndrome subtype sensitivity analysis was performed. Therefore, possible immortal-time bias, treatment-selection bias, and subtype heterogeneity were considered when interpreting the findings. No formal multiplicity adjustment was applied to secondary outcomes; therefore, their p-values were interpreted as exploratory. A p-value ≤0.05 was considered statistically significant.

Ethical considerations

Ethical approval was obtained from the Institutional Review Board/Ethics Review Committee of Lahore University of Biological and Applied Sciences (UBAS), a project of Lahore Medical and Dental College (LMDC), Lahore, Pakistan, under approval No. ERC/2025/003A. The requirement for individual informed consent was waived because the study involved retrospective analysis of anonymized medical records. Patient confidentiality was maintained in accordance with institutional ethical requirements and the principles of the Declaration of Helsinki.

## Results

Baseline characteristics of the study population

The study included 200 patients diagnosed with acute coronary syndrome, with 100 patients in the accelerated-pathway group and 100 in the standard-care group. Patients were classified according to the timing of documented definitive management and were not randomly assigned. The mean age was 59.2 ± 10.8 years in the accelerated-pathway group and 60.1 ± 11.3 years in the standard-care group. Male patients accounted for 64.0% and 61.0% of the groups, respectively.

No statistically significant between-group differences were observed in the measured baseline demographic characteristics, cardiovascular risk factors, comorbidities, or acute coronary syndrome subtypes. Non-ST-segment elevation myocardial infarction was the most frequent subtype in both groups, followed by ST-segment elevation myocardial infarction and unstable angina, as shown in Table 1.

**Table 1:** Baseline demographic and clinical characteristics of patients with acute coronary syndrome NSTEMI: non-ST-segment elevation myocardial infarction; STEMI: ST-segment elevation myocardial infarction.

Variable	Accelerated-pathway group, n=100	Standard-care group, n=100	p-value
Age, years	59.2 ± 10.8	60.1 ± 11.3	0.560
Male sex, n (%)	64 (64.0)	61 (61.0)	0.660
Female sex, n (%)	36 (36.0)	39 (39.0)	0.660
Hypertension, n (%)	63 (63.0)	67 (67.0)	0.550
Diabetes mellitus, n (%)	45 (45.0)	49 (49.0)	0.570
Smoking history, n (%)	41 (41.0)	38 (38.0)	0.660
Dyslipidemia, n (%)	39 (39.0)	42 (42.0)	0.670
Previous ischemic heart disease, n (%)	26 (26.0)	29 (29.0)	0.630
Chronic kidney disease, n (%)	12 (12.0)	15 (15.0)	0.530
STEMI, n (%)	34 (34.0)	31 (31.0)	0.650
NSTEMI, n (%)	48 (48.0)	51 (51.0)	0.670
Unstable angina, n (%)	18 (18.0)	18 (18.0)	1.000

High-sensitivity troponin testing and early clinical management

High-sensitivity cardiac troponin testing was documented at presentation for all included patients. Initial troponin positivity occurred in 74.0% of patients in the accelerated-pathway group and 71.0% in the standard-care group. A dynamic rise or fall in serial troponin concentrations was documented in 68.0% and 64.0% of patients, respectively. Neither difference was statistically significant. The mean time from hospital presentation to definitive clinical decision was shorter in the accelerated-pathway group than in the standard-care group (2.1 ± 0.6 vs. 5.7 ± 1.8 hours). The mean between-group difference was −3.60 hours (95% confidence interval: −3.97 to −3.23; p<0.001). Patients in the accelerated-pathway group more frequently received early cardiology assessment, monitored-unit admission, coronary angiography within 24 hours, dual antiplatelet therapy, anticoagulation where indicated, and high-intensity statin therapy. Treatment timing and management patterns are presented in Table 2.

**Table 2:** High-sensitivity troponin testing, treatment timing, and management pattern hs-cTn: high-sensitivity cardiac troponin. Absolute differences for categorical variables were calculated as the accelerated-pathway percentage minus the standard-care percentage.

Variable	Accelerated-pathway group, n=100	Standard-care group, n=100	Absolute difference (95% CI)	p-value
Initial hs-cTn positive, n (%)	74 (74.0)	71 (71.0)	3.0 percentage points (−9.4 to 15.4)	0.630
Dynamic hs-cTn rise/fall, n (%)	68 (68.0)	64 (64.0)	4.0 percentage points (−9.1 to 17.1)	0.550
Time to definitive clinical decision, hours	2.1 ± 0.6	5.7 ± 1.8	−3.60 hours (−3.97 to −3.23)	<0.001
Early cardiology assessment, n (%)	91 (91.0)	73 (73.0)	18.0 percentage points (7.6 to 28.4)	0.001
Monitored-unit admission, n (%)	86 (86.0)	69 (69.0)	17.0 percentage points (5.7 to 28.3)	0.004
Coronary angiography within 24 hours, n (%)	59 (59.0)	41 (41.0)	18.0 percentage points (4.4 to 31.6)	0.011
Dual antiplatelet therapy, n (%)	94 (94.0)	84 (84.0)	10.0 percentage points (1.4 to 18.6)	0.024
Anticoagulation where indicated, n (%)	89 (89.0)	78 (78.0)	11.0 percentage points (0.8 to 21.2)	0.037
High-intensity statin therapy, n (%)	96 (96.0)	87 (87.0)	9.0 percentage points (1.4 to 16.6)	0.025

Mortality and intensive care outcomes

The primary outcome, 30-day all-cause mortality, occurred in 7 patients in the accelerated-pathway group and 16 patients in the standard-care group, corresponding to rates of 7.0% and 16.0%, respectively. The absolute risk difference was −9.0 percentage points (95% confidence interval: −17.8 to −0.2; p=0.046). In-hospital mortality occurred in 5.0% of patients in the accelerated-pathway group and 11.0% in the standard-care group; this difference was not statistically significant. Recurrent myocardial infarction occurred in 6.0% and 14.0% of patients, respectively, and also did not reach statistical significance.

Acute heart failure occurred in 11.0% of patients in the accelerated-pathway group and 24.0% in the standard-care group. Malignant arrhythmias occurred in 8.0% and 18.0%, cardiogenic shock in 6.0% and 15.0%, and intensive care unit admission in 14.0% and 28.0%, respectively. These secondary outcomes were less frequent in the accelerated-pathway group in the unadjusted analyses. The mean hospital stay was 4.7 ± 1.5 days in the accelerated-pathway group and 6.3 ± 2.2 days in the standard-care group, with a mean difference of −1.60 days (95% confidence interval: −2.12 to −1.08; p<0.001). Detailed outcomes are presented in Table 3. Because no formal multiplicity adjustment was applied, all secondary-outcome analyses were considered exploratory.

**Table 3:** Mortality, clinical complications, and intensive care outcomes CI: confidence interval.

Outcome	Accelerated-pathway group, n=100	Standard-care group, n=100	Crude absolute difference (95% CI)	p-value
In-hospital mortality, n (%)	5 (5.0)	11 (11.0)	−6.0 percentage points (−13.5 to 1.5)	0.120
30-day mortality, n (%)	7 (7.0)	16 (16.0)	−9.0 percentage points (−17.8 to −0.2)	0.046
Recurrent myocardial infarction, n (%)	6 (6.0)	14 (14.0)	−8.0 percentage points (−16.2 to 0.2)	0.058
Acute heart failure, n (%)	11 (11.0)	24 (24.0)	−13.0 percentage points (−23.4 to −2.6)	0.015
Malignant arrhythmia, n (%)	8 (8.0)	18 (18.0)	−10.0 percentage points (−19.2 to −0.8)	0.035
Cardiogenic shock, n (%)	6 (6.0)	15 (15.0)	−9.0 percentage points (−17.4 to −0.6)	0.037
Intensive care unit admission, n (%)	14 (14.0)	28 (28.0)	−14.0 percentage points (−25.1 to −2.9)	0.015
Hospital stay, days	4.7 ± 1.5	6.3 ± 2.2	−1.60 days (−2.12 to −1.08)	<0.001

Predictors of 30-day mortality

An exploratory multivariable logistic regression analysis was performed to examine factors associated with 30-day mortality. A total of 23 deaths occurred among the 200 patients. Because eight variables were included despite the limited number of events, the adjusted estimates were considered potentially unstable and susceptible to model overfitting.

Accelerated-pathway management was associated with lower adjusted odds of 30-day mortality (adjusted odds ratio: 0.40; 95% confidence interval: 0.16-0.98; p=0.045). Diabetes mellitus was associated with higher adjusted odds of mortality (adjusted odds ratio: 2.12; 95% confidence interval: 1.03-4.93; p=0.041). Killip class III-IV, delayed presentation beyond six hours, and cardiogenic shock also showed higher adjusted odds of mortality.

Age above 65 years, hypertension, and ST-segment elevation myocardial infarction presentation had adjusted odds ratios above 1.0, but their confidence intervals included the null value, and the associations were not statistically significant, as shown in Table 4. The regression findings were interpreted as exploratory associations rather than definitive independent or causal effects.

**Table 4:** Multivariable logistic regression analysis for predictors of 30-day mortality hs-cTn: high-sensitivity cardiac troponin; STEMI: ST-segment elevation myocardial infarction.

Variable	Adjusted odds ratio	95% confidence interval	p-value
Accelerated hs-cTn-anchored care pathway	0.40	0.16-0.98	0.045
Age above 65 years	1.68	0.79-3.58	0.180
Diabetes mellitus	2.12	1.03-4.93	0.041
Hypertension	1.31	0.62-2.78	0.470
STEMI presentation	1.82	0.86-3.91	0.110
Killip class III-IV	3.74	1.58-8.86	0.003
Delayed presentation >6 hours	2.69	1.19-6.08	0.018
Cardiogenic shock	4.86	1.91-12.36	0.001

## Discussion

This retrospective observational cohort study evaluated the association between an accelerated high-sensitivity cardiac troponin-anchored care pathway and short-term outcomes in patients with acute coronary syndrome [1]. Compared with standard care, the accelerated pathway was associated with faster clinical decision-making, lower observed 30-day mortality, fewer cardiovascular complications, reduced intensive care unit admission, and shorter hospital stay. However, because treatment allocation was not randomized, these findings represent associations and do not establish causality [2].

Thirty-day mortality was 7.0% in the accelerated-pathway group and 16.0% in the standard-care group [3]. This difference may reflect the earlier translation of clinical, electrocardiographic, and biomarker findings into definitive management. Persistent myocardial ischemia may lead to infarct extension, left ventricular dysfunction, malignant arrhythmias, cardiogenic shock, and death. Therefore, reducing delays in specialist assessment, monitoring, medical treatment, and invasive evaluation may be clinically important [4-6].

Initial troponin positivity and dynamic serial changes were similar between the groups, whereas the time to definitive clinical decision was substantially shorter in the accelerated-pathway group [7]. Patients in this group also more frequently received early cardiology assessment, monitored-unit admission, coronary angiography within 24 hours, dual antiplatelet therapy, anticoagulation where indicated, and high-intensity statin therapy. The exposure therefore represented a coordinated acute coronary syndrome care pathway rather than the isolated effect of troponin testing. High-sensitivity troponin should be interpreted alongside symptoms, electrocardiographic findings, serial biomarker changes, renal function, hemodynamic status, and overall clinical risk [8].

Acute heart failure, malignant arrhythmias, cardiogenic shock, and intensive care unit admission occurred less frequently in the accelerated-pathway group [9,10]. Hospital stay was also shorter. Earlier assessment and treatment may reduce diagnostic uncertainty and clinical deterioration while facilitating stabilization and discharge [11]. Nevertheless, these outcomes were secondary and exploratory, and no formal adjustment for multiple comparisons was performed. The study was also not designed to assess cost-effectiveness, bed utilization, or healthcare resource allocation; therefore, system-level benefits require confirmation in dedicated health-service studies [12].

Exploratory multivariable analysis showed an association between accelerated-pathway management and lower adjusted odds of 30-day mortality [13]. Diabetes mellitus, Killip class III-IV, delayed presentation beyond six hours, and cardiogenic shock were associated with higher adjusted odds of mortality. However, only 23 deaths occurred, while eight predictors were included in the model. The estimates may therefore be unstable because of overfitting and should not be regarded as definitive independent or causal effects [14].

The study contributes by examining mortality and intensive care-related outcomes within an accelerated high-sensitivity troponin-anchored pathway in routine clinical practice [15]. Previous studies have largely focused on diagnostic performance, rule-in or rule-out algorithms, or risk stratification. The present findings instead highlight the potential importance of promptly integrating biomarker results into coordinated clinical management [16].

Several limitations should be considered. The retrospective, non-randomized design was susceptible to incomplete documentation, selection bias, confounding by indication, treatment-selection bias, and residual confounding [17]. Classification according to management within three hours may also have introduced immortal-time bias, and no landmark or early-death sensitivity analysis was performed. The limited sample size and mortality event count increased the risk of regression-model overfitting. Chronic kidney disease was not included in the adjusted model, and complete left ventricular ejection fraction data were unavailable [18].

Including ST-segment elevation myocardial infarction, non-ST-segment elevation myocardial infarction, and unstable angina introduced clinical heterogeneity because troponin has different diagnostic roles across these subtypes [19,20]. No subtype-specific analysis was conducted. Multiple secondary outcomes were evaluated without multiplicity adjustment, and outcomes were not independently adjudicated. Unmeasured social determinants may also have influenced presentation and treatment timing. Finally, the single-center setting and 30-day follow-up limit generalizability and assessment of longer-term outcomes. Larger multicenter studies using standardized adjudication, appropriate adjustment, and sensitivity analyses are required [21,22].

## Conclusions

In this retrospective observational cohort, management through an accelerated high-sensitivity cardiac troponin-anchored acute coronary syndrome care pathway was associated with lower observed 30-day mortality, fewer cardiovascular complications, reduced intensive care unit admissions, and shorter hospital stays. These findings reflect the broader coordinated care pathway rather than the isolated effect of troponin testing. Because of the non-randomized design, limited event count, possible immortal-time and treatment-selection bias, subtype heterogeneity, and residual confounding, causal conclusions cannot be drawn. Larger multicenter studies with robust adjustment, standardized outcome adjudication, and appropriate sensitivity analyses are needed to confirm these findings.
